# The importance of boundary evolution for solar-wind modelling

**DOI:** 10.1038/s41598-024-80162-2

**Published:** 2024-11-22

**Authors:** Mathew J. Owens, Luke Barnard, Charles N. Arge

**Affiliations:** 1 Department of Meteorology, University of Reading, Earley Gate, Reading, Berkshire RG6 6BB UK; 2 Solar Physics Laboratory, NASA/GSFC, Mail Code 671, Greenbelt, MD 20771 USA

**Keywords:** Space physics, Solar physics, Space physics, Solar physics

## Abstract

The solar wind is a continual outflow of plasma and magnetic field from the Sun’s upper atmosphere—the corona—that expands to fills the solar system. Variability in the near-Earth solar-wind conditions can produce adverse space weather that impacts ground- and space-based technologies. Consequently, numerical fluid models of the solar wind are used to forecast conditions a few days ahead. The solar-wind inner-boundary conditions are supplied by models of the corona that are, in turn, constrained by observations of the photospheric magnetic field. While solar eruptions—coronal mass ejections (CMEs)—are treated as time-dependent structures, a single coronal “snapshot” is typically used to determine the ambient solar-wind for a complete model run. Thus, all available time-history information from previous coronal-model solutions is discarded and the solar wind is treated as a steady-state flow, unchanging in the rotating frame of the Sun. In this study, we use 1 year of daily-updated coronal-model solutions to comprehensively compare steady-state solar-wind modelling with a time-dependent method. We demonstrate, for the first time, how the SS approach can fundamentally misrepresent the accuracy of coronal models. We also attribute three key problems with current space-weather forecasting directly to the steady-state approach: (1) the seemingly paradoxical result that forecasts based on observations from 3-days previous are more accurate than forecasts based on the most recent observations; (2) high inconsistency, with forecasts for a given day jumping significantly as new observations become available, changing CME propagation times by up to 17 h; and (3) insufficient variability in the heliospheric magnetic field, which controls solar energetic particle propagation to Earth. The time-dependent approach is shown to alleviate all three issues. It provides a consistent, physical solution which more accurately represents the information present in the coronal models. By incorporating the time history in the solar wind along the Sun-Earth line, the time-dependent approach will provide improvements to forecasting CME propagation to Earth.

## Introduction

Space weather—variability in the conditions in the near-Earth space environment—can have adverse effects on a range of ground- and space-based technologies^1,2^. The most extreme space weather is driven by transient coronal mass ejections (CMEs)^3^, rather than the continually out-flowing ‘ambient’ solar wind^4^. But in order to understand and model the propagation of CMEs to Earth, and therefore to forecast space-weather hazards with more than the 1-h warning from in situ data collected near 1 AU, it is necessary to accurately reconstruct ambient solar-wind conditions between the Sun to the Earth^5^.

The large-scale solar-wind flow can be well represented by the magnetohydrodynamic (MHD) approximation^6^. A number of numerical, 3-dimensional, MHD models have been developed for solar-wind science and forecasting^7–11^. These are now standard tools for both research and operations, but there is an issue with how they are currently used.

Heliospheric models of the solar wind typically have an inner boundary around 0.1 AU, where the solar-wind flow is supersonic, meaning information only propagates outwards. The inner boundary conditions are provided by coronal models, themselves constrained by photospheric magnetic field observations. Almost universally, a single steady-state (SS) coronal-model solution is used for the entire duration of a (heliospherc) solar-wind model run, normally 3–5 days to allow CMEs to propagate through the model domain to Earth. Thus, in the rotating frame of the Sun, the solar-wind inner-boundary conditions are unchanging and the ambient solar wind is treated as SS flow. Into this SS ambient flow, time-dependent perturbations are added to mimic the passage of CMEs^12,13^. All current operational solar-wind forecast models operate in this SS fashion and have done for 20 years^14,15^. Most scientific studies of the heliospheric structure and CME propagation follow the same SS framework for the ambient solar wind, even when the CME eruption is obtained through fully time-dependent coronal modelling^16,17^.

One of the reasons that this SS approach has become the *defacto* standard is likely because coronal models—at least in operational forecasting—are typically steady-state ‘snapshots’ themselves, owing to either observational or computational limitations^18^. Such coronal snapshots, however, are updated with a daily (or faster) cadence, as new photospheric magnetic field observations become available. As this time-scale is faster than the solar-wind propagation time to Earth, even SS coronal models do provide useful information about time-evolution of the solar-wind inner-boundary conditions. The principle of exploiting this time-history information for solar-wind modelling has been demonstrated: Coronal snapshots have been used to produce quasi-time-dependent (TD) inner-boundary conditions to solar-wind models that generate solar-wind structures and heliospheric magnetic field features in qualitative agreement with observations^10,19^. But this approach is not routinely used for either science of forecasting, presumably as the benefits over the SS approach have not been quantitatively demonstrated.

Instead, each solar-wind solution makes use of only a single coronal ‘snapshot’, discarding the potentially valuable time-history information. As solar-wind models are the intermediary by which coronal models can be confronted with near-Earth solar wind observations^20–22^, the SS approximation is the lens through which coronal models are currently validated. Thus any issues arising from SS solar-wind modelling will misrepresent coronal-model performance and potentially lead to regressive coronal-model development. The aim of this study is fully assess the implications of the SS solar-wind approach and test whether these are alleviated by a TD approach.

**Fig. 1:**
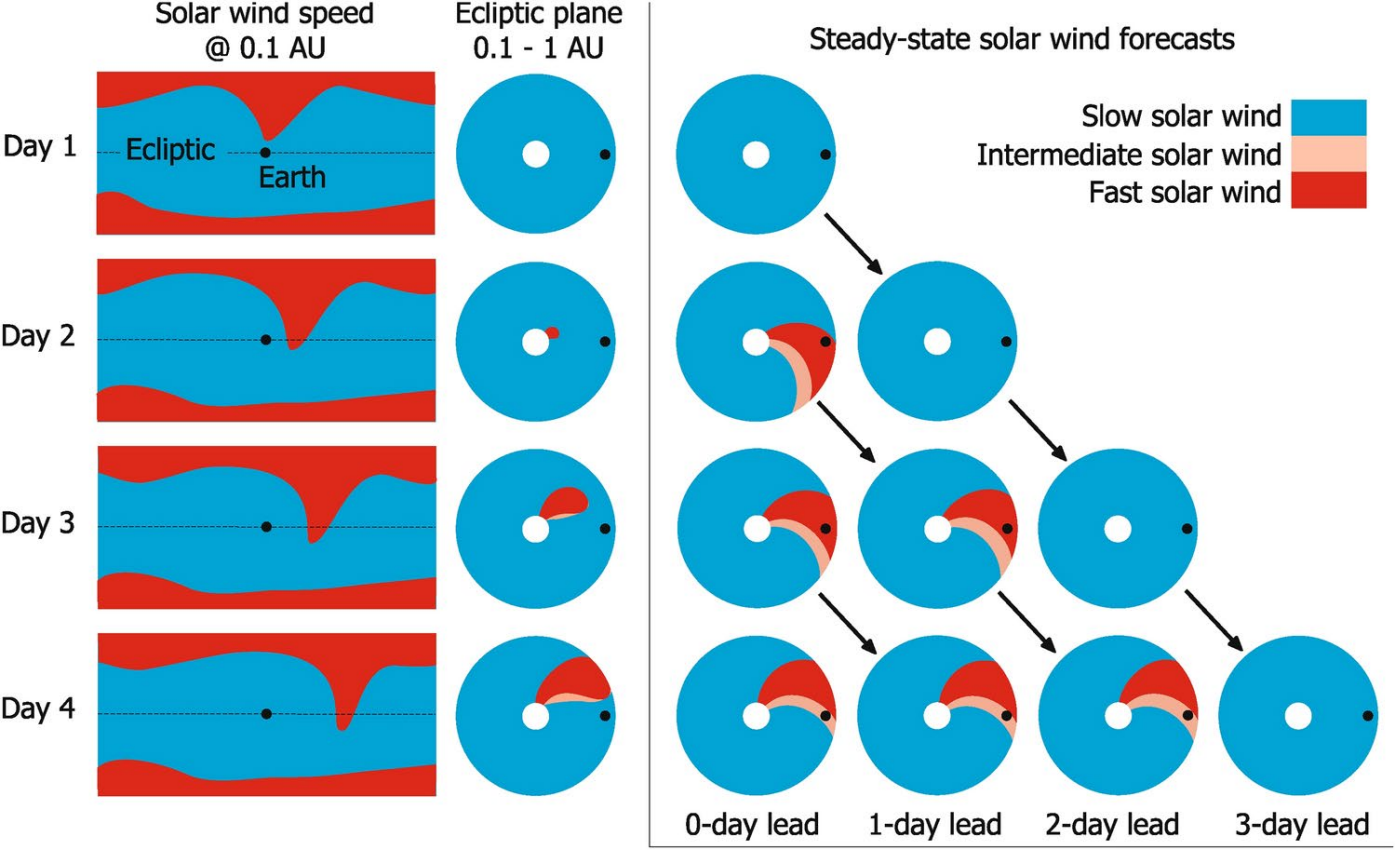
A schematic of the construction of a steady-state solar-wind forecast. Rows, from top to bottom, show consecutive days. The left column shows solar-wind speed on latitude-longitude maps at the top of the corona (e.g. 0.1 AU), such as is produced by a coronal model. The Earth is shown by the black dot and the ecliptic plane by the horizontal dashed line. Red and blue indicate fast and slow wind, respectively. The middle column shows the resulting solar wind speed structure in the ecliptic plane from 0.1 to 1 AU, as a fast stream emerges and creates intermediate-speed wind (pink) behind it. The panels in the box show the solar-wind speed structure in the ecliptic from a steady-state model, and how they are used to produce forecasts of different lead times. See text for further discussion.

Before comparing SS and TD approaches from genuine coronal-model output, it is useful to work through a hypothetical example to show how a forecast is constructed in the SS framework and how the implications of the SS assumption change with different forecast lead times (i.e. whether we are considering the solar-wind conditions being forecast for 1, 2, 3... days in the future). The left-hand panels of Fig. 1 show typical solar-wind conditions at 0.1 AU around the time of solar minimum: Fast wind at the poles, with slow wind at the equator. There is a fast-wind extension from the north pole to lower latitudes which rotates around in longitude with the Sun. The fast-wind extension is also evolving in time, and arrives at the ecliptic plane on day 2 and persists through day 4. This means that in reality, there is no fast wind anywhere in the ecliptic plane out to Earth orbit on day 1 and a fast stream then propagates radially outward from day 2, reaching 1 AU on day 4. As the Sun rotates, this propagating fast stream forms into a Parker-spiral^23^ structure, with intermediate-speed wind in the rarefaction region behind the fast wind. However, as the fast-stream extension was at no time located at the sub-Earth point, neither the fast- nor intermediate-speed winds encounter Earth (assuming radial flow) at any time. Thus, the true solar-wind state resulting from these 0.1-AU boundary conditions is slow wind in near-Earth space for all 4 days.

The left-hand panels of Fig. 1 show the result of applying SS solar-wind modelling to the same 0.1-AU boundary conditions. On day 1, the SS model correctly reconstructs slow wind throughout the ecliptic plane. This day 1 reconstruction becomes the 1-day lead-time forecast for day 2, the 2-day lead-time forecast for day 3, and the 3-day lead-time forecast for day 4.

On day 2, however, the newly-arrived fast wind in the ecliptic causes issues when treated as a SS phenomenon. The SS model only has access to the current boundary conditions and must therefore assume they have persisted indefinitely. Therefore instead of a new fast stream physically propagating radially outward through the model domain over the next 3 days, it instead instantaneously appears at 1 AU, incorrectly engulfing the Earth in fast wind. This (incorrect) SS solution is rotated with the Sun to become the 1-day lead-time forecast for day 3, and the 2-day lead-time forecast for day 4. A similar situation occurs for SS forecasts made on days 3 and 4.

The observations at Earth for this 4-day interval would show slow wind throughout. Thus, the net result is that on day 4, the most accurate forecast is not the most recent one, but that produced 3 days previously. Crucially, that 3-day lead-time forecast was based on the state of the Sun when the solar wind began its journey to Earth.

**Fig. 2:**
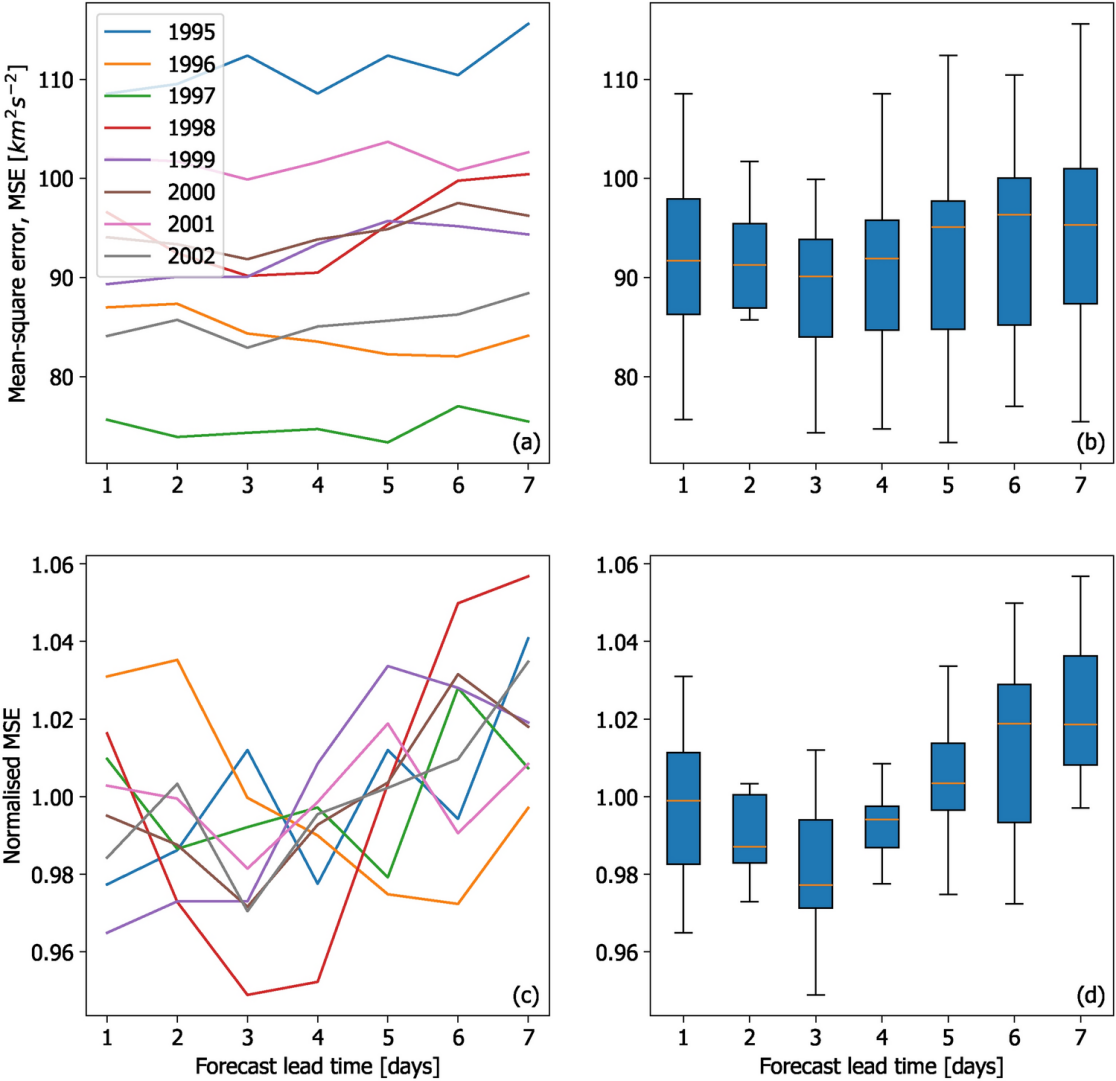
A summary of the WSA model solar-wind speed forecasts presented by^24^. (**a**) Mean-square error (MSE) of forecasts for 1995–2002 as a function of forecast lead time. (**b**) The same data presented as a box-and-whiskers plot. The orange line shows the median MSE over all years. The blue bars span the interquartile range, while the black lines span the full range. (**c**) The same data as panel a, but normalised by the average MSE for each year. (**d**) The same normalised data presented as a box-and-whiskers plot.

This effect has been reported, at least in passing, in previous solar-wind forecast-validation studies: Fig. 2 shows a summary of the results of solar-wind speed forecasts for the period 1995–2002, as originally presented in Table 1 of^24^. Panel a shows the mean-square error (MSE) between the forecast solar-wind speed at Earth and that observed. I.e.

1 $$\begin{aligned} \hbox {MSE} = \frac{1}{N}\sum _{t=1}^{N}\left( V_F^t - V_O^t\right) ^2, \end{aligned}$$

where *N* is the total number of time steps considered, $V_F^t$ is the forecast solar wind speed at time *t*, and $V_O^t$ is the observed solar wind speed at time *t*.

The MSE is dominated by the inter-annual variability, likely linked to the solar-cycle variation in coronal and solar-wind structure. This makes it difficult to immediately see the effect of forecast lead time. Nevertheless, even taking a simple average of MSE over all years (panel b) shows that the error is indeed lowest for 3-day lead times. But this becomes clearer still when MSE is normalised by the mean MSE for each year, to remove any solar cycle trend, as shown by panels c and d. The 3-day lead-time forecast is now statistically better than the 1-day lead-time forecast, consistent with the hypothetical example shown in Fig. 1.

These data—and results presented in the current study—argue strongly that the SS approach is inherently problematic. The remainder of the paper compares the results of SS and TD solar-wind modelling for identical boundary conditions, generated on a daily basis for the whole of 2020. Section “Methods” presents the data and models used to perform the experiments. Section “A case study: December 2020” presents a case study from December 2020, which shows a number of similar features to the hypothetical example shown in Fig. 1. A statistical analysis of forecast accuracy, forecast consistency and the heliospheric magnetic field (HMF) orientation is then presented over the whole 2020 interval in Section “Statistical analysis”. The implications of these findings and future directions are discussed in Section “Discussion”.

## Methods

### Data

The SS and TD solar-wind models are tested using 1 year of daily-updated coronal model solutions. As the aim of this study is not to test the coronal models *per se*, we select 2020, a year close to solar minimum, when the coronal models should perform reasonably well at a global scale. It also means the solar-wind structure is most slowly evolving^25^, providing the best conditions for the SS assumption. However, the slow evolution of the corona does not necessarily mean that the solar wind will be well forecast at this time, as the flat heliospheric current sheet means small positional uncertainties in the coronal solution can lead to big errors in the reconstructed solar-wind speed^24^.

The inner-boundary conditions to the coronal model are the Global Oscillation Network Group (GONG) photospheric magnetic field observations, made from ground-based observatories^26^. The Earth-facing solar-disc observations are used to produce complete maps of the whole photosphere as the Sun rotates, which is here updated on a daily basis. The update brings in new information both of the magnetic field structure on the nearside of the Sun which has changed from the previous day, and from new longitudes on the west limb that rotate into view.

In order to reduce the effect of observations being limited to the Earth-facing portion of the photosphere at any one time, we use GONG photospheric maps constructed by the Air Force Data Assimilative Photospheric Flux Transport (ADAPT) system. This evolves the photospheric magnetic field using known photospheric flows^27,28^. ADAPT attempts to produce synchronic maps which better represent the global photosphere at a given moment. As flux transport is a quasi-stochastic process, it involves intrinsic uncertainty. Thus, ADAPT produces an ensemble of 12 solutions for each time step (in this study, each day). By minimising the discontinuities in the photospheric boundary as a function of time, the use of ADAPT again provides the best-possible conditions for SS solar-wind models.

The daily-updated GONG-ADAPT synchronic maps are used as boundary conditions for the Wang-Sheeley-Arge (WSA) coronal model^29^. The WSA model is based on the potential-field source-surface (PFSS) approximation^30^ and an empirical method to determine the radial solar-wind flow speed, *V*, at the outer boundary. As such, it is itself a SS coronal model. The WSA output is an estimate of the radial magnetic field, $B_R$, and *V*, typically at 0.1 AU, where they serve as the inner-boundary conditions to solar-wind models. WSA does not directly provide an estimate of plasma density or temperature. We here limit analysis to *V*, which is of most immediate space-weather concern, as it directly affects the arrival time of CMEs.

**Fig. 3:**
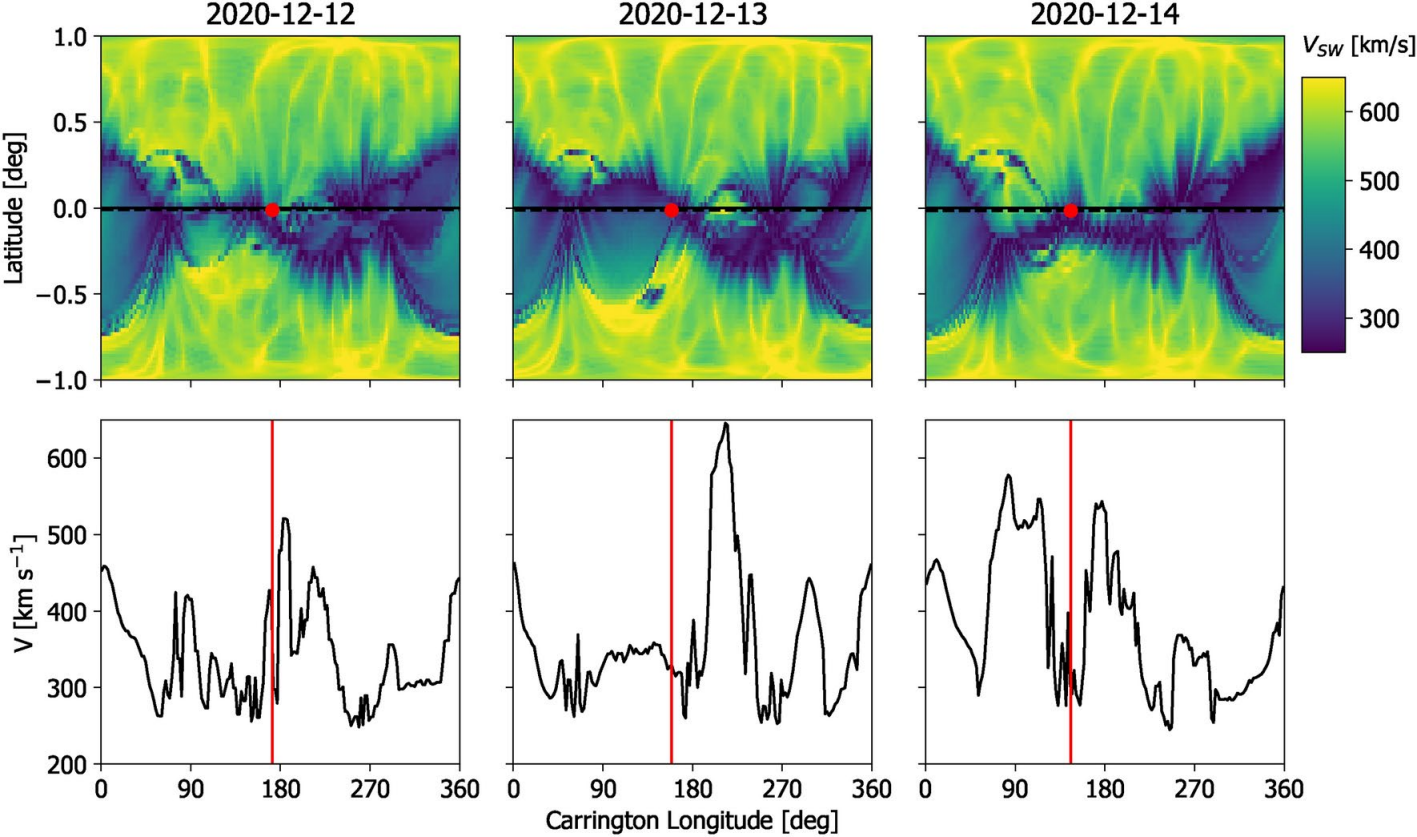
Examples of the WSA solar-wind speed, *V*, solutions at 0.1 AU—the inner boundary to the solar-wind models—for three consecutive days in December 2020. For simplicity, only results from the first of twelve ADAPT reasliations are shown. Top: *V* as a function of Carrington longitude and the sine of heliographic latitude. The dashed line shows Earth latitude, with the red dot indicating the sub-Earth point. Bottom: *V* at Earth latitude, as a function of Carrington longitude. Note the appearance and disappearance of a high-speed stream just ahead of Earth in its orbit, around 200–230 degrees Carrington longitude.

Figure 3 shows examples of WSA-GONG-ADAPT *V* at 0.1 AU for three consecutive days in December 2020. Only the first of twelve ADAPT ensemble members is shown. As can be seen in the top panels, this is a fairly typical solar minimum configuration, with fast wind at high latitudes and slow wind confined to a band centred on the helio-equator. At the global scale, the solution does not change substantially over the 3 days. However, looking at the details of *V* at Earth latitude (the bottom panels), differences are readily apparent, particularly around 200-degrees Carrington longitude, just ahead of Earth in its orbit at this time. On 2020-12-12, there is slow and intermediate speed wind at this location. On 2020-12-13, fast wind appears, before disappearing/declining again on 2020-12-14. This large change in *V* at Earth latitude, despite very small global coronal changes, is symptomatic of the flat heliospheric current sheet at this time.

Each of these WSA solutions is steady state, but this series of SS solutions implies rapid time variation of *V* in the ecliptic plane. The consequences of this are investigated in Section “A case study: December 2020”. Whether this particular short-lived fast stream is ‘real’ or not is a moot point for the purposes of thus study; the coronal structure does evolve on these kinds of time scales and thus there is a need to determine the implications of this time evolution when used with the current SS approach.

**Fig. 4:**
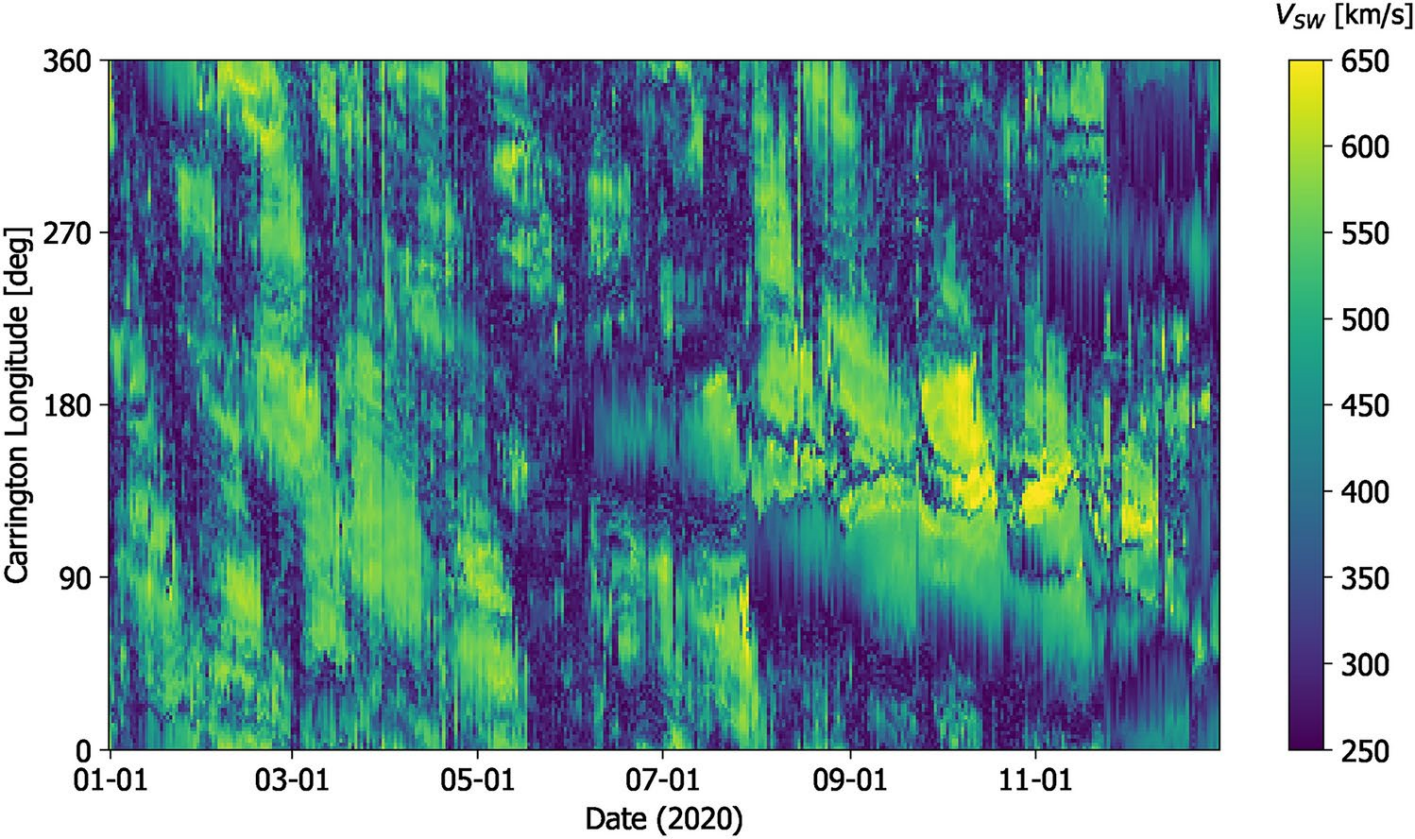
Solar-wind speeds at 0.1 AU and Earth latitude, as a function of Carrington longitude and time. This solution is for a single WSA-ADAPT-GONG realisation for the year 2020.

Figure 4 shows *V* at Earth latitude over the whole 2020 interval from daily-updated WSA-GONG-ADAPT estimates. Only the first ADAPT ensemble member is shown, but the others are qualitatively similar. Approximately 27-day banded structure can be seen. These quasi-discontinuous changes in the WSA solution result when active regions rotate onto the near-side of the Sun and the GONG-ADAPT synchronic map—and hence WSA solution—is updated.

While the aim of the study is not to validate the corona model solutions themselves, we do compare the solar-wind model output to the near-Earth in situ observations of *V*. For this purpose, we use the OMNI database of near-Earth solar wind observations^31^, averaged to 1-h resolution.

### Solar-wind modelling

For each day in 2020, we have 12 WSA solutions, one for each GONG-ADAPT realisation of the photospheric synchronic map. A solar-wind model must then be run twice for each of these 12 realisations—once with SS boundary conditions and once with TD boundary conditions. Each solar-wind model run is 40 days long, to provide comparison of the short forecast lead times with the recurrent nature of the solar wind (i.e. forecasts 27-days ahead). Thus, the study involves solar-wind model runs total around 350,000 days of simulated time.

Three-dimensional MHD solar wind models, such as EUHFORIA, typically require around 30 min per simulated day on 128 CPU cores (Poedts, personal communication). Consequently, this study would need of the order $10^7$ core hours to complete using 3DMHD solar-wind models, which is prohibitive with current computational resources. Instead, we use a surrogate model for 3DMHD, the Heliospheric Upwind eXatrpolation model with time dependence [HUXt^32,33^]. HUXt is a dynamical model that treats the solar wind as a 1-dimensional, incompressible hydrodynamic flow [see also^34^]. Thus, solar-wind flows may accelerate and decelerate through stream interaction, but only solar wind speed, not density or temperature, is reconstructed. Despite the gross approximations, HUXt produces *V* throughout the model domain from 0.1 to 1 AU that agrees very closely with 3DMHD for the same boundary conditions^32^, but at a fraction of the computational cost. HUXt allows us to complete this study using less than 10 core hours, while results are still expected to be illustrative of 3DMHD.

For each of the 12 WSA-GONG-ADAPT solutions per day, we extract *V* at 0.1 AU at Earth latitude. We first use this to generate a SS solution; driving HUXt with constant inner-boundary conditions for 40 days. No information from previous days’ WSA solutions is used.

Next, for each of the 12 WSA-GONG-ADAPT solutions per day, we also produce a TD solution. These take *V* at Earth latitude from the WSA-GONG-ADAPT solutions from the previous 5 days. As the solar-wind travel time from the model inner boundary at 21.5 $r_S$ to outer boundary at 215 $r_S$ is approximately 2–4 days, there is no advantage to starting the model run more than 5 days prior to the time the forecast is issued, as the information will have completely propagated out of the model domain. The inner boundary condition at a given time is produced by linear interpolation between WSA-GONG-ADAPT solutions on consecutive days; while more sophisticated treatments of the boundary conditions are possible^35^, as will be shown, this nevertheless provides an adequate starting point. As these model runs are hindcasts, there are no new WSA-GONG-ADAPT solutions to use after the forecast issue date, so the HUXt inner-boundary conditions are held constant at the most recent WSA-GONG-ADAPT solution. Thus on day 1, the model domain will contain information from the previous few days of WSA-GONG-ADAPT solutions, but by day 4, the TD boundary-conditions will have propagated out of the model domain and the solution will revert to a SS solution.

For both the SS and TD runs, each individual model run is divided up to provide forecasts at a range of lead times. Specifically, each individual 40-day run is used up to produce 24 h of 1- through 40-day advance forecasts, as shown in Fig. 1. By combining multiple runs, we produce 1- to 40-day advance forecasts over the whole 2020 interval. If forecasts produced from 1 day to the next vary substantially, this will result in discontinuities in the forecast time series of a given lead time.

Each forecast time series is compared with the observed *V* at Earth. All data are averaged to 1-h resolution and the mean-absolute error (MAE) is computed between the forecast and observation:

2 $$\begin{aligned} \hbox {MAE} = \frac{1}{N}\sum _{t=1}^{N} \left| V_F^t - V_O^t\right| , \end{aligned}$$

where *N* is the total number of time steps considered, $V_F^t$ is the forecast solar wind speed at time *t*, and $V_O^t$ is the observed solar wind speed at time *t*. While more sophisticated metrics are useful when comparing qualitatively different forecasts^36^, in this instance, the SS and TD time series are qualitatively similar and MAE provides reasonable insight into relative performance.

## Results

### A case study: December 2020

Before showing the statistical analysis of the full 2020 dataset, it is instructive to look in detail at an example period. We use December 2020, as this features a number of fast streams in both the WSA-GONG-ADAPT results and the observed near-Earth *V*. For simplicity, we only show results from the first ADAPT realisation, though the subsequent statistical analysis uses all twelve ensemble members.

**Fig. 5:**
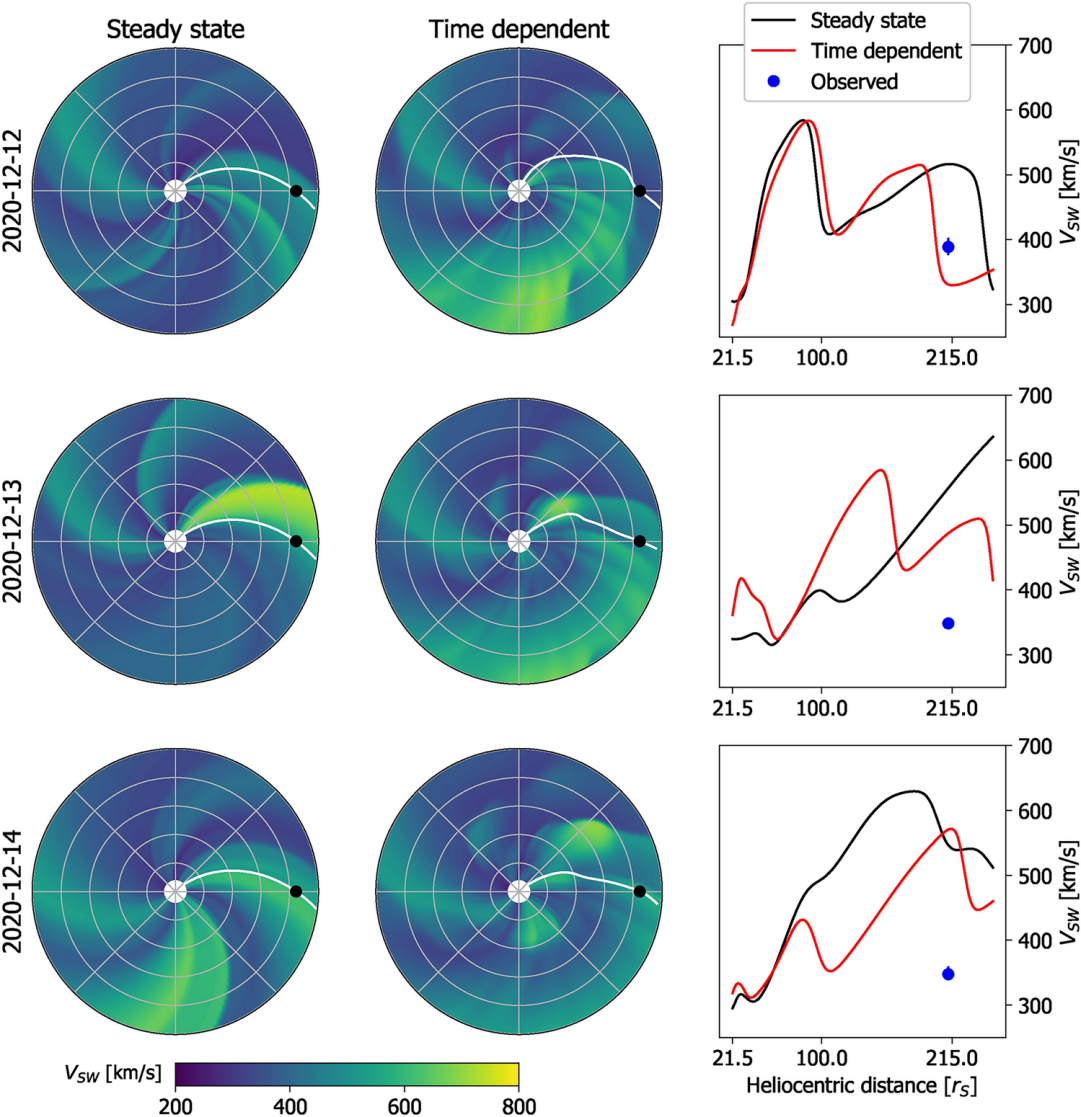
Examples of HUXt solutions to the WSA-GONG-ADAPT *V* for the three consecutive days in December 2020 shown in Fig. 3. Left: *V* obtained by the SS solar-wind solutions in the ecliptic plane, using the HUXt solar-wind model. The Earth is shown as the black dot and the Earth-connected magnetic-field line is shown in white. Middle: The time-dependent solar wind solutions using the HUXt model, in the same format. Right: *V* along the Earth-Sun line for the SS (black) and TD (red) solutions. The observed solar wind speed in near-Earth space is shown by the blue dot. An animated version of this figure is available in the supplementary information.

Figure 5 shows the TD and SS HUXt solutions from HUXt to the same 3 days in December 2020 that were shown in Fig. 3. This figure can also be viewed as a movie, which makes the features under discussion much easier to identify. For this period, neither the SS nor the TD modelling produces a particularly good match to the observed *V*: forecast accuracy is primarily determined by the quality of the inner-boundary conditions provided by the coronal model. But this period nevertheless highlights important differences in the forecast which result from how the exact same inner-boundary conditions are treated. As highlighted in Fig. 3, on 2024-12-13 a fast stream appears at 0.1 AU at a longitude ahead of Earth, with respect to solar rotation. The fast stream has mostly disappeared again by 2024-12-14. Providing the full TD boundary conditions to the HUXt model means this short burst of fast wind effectively produces a localised *V* transient that moves radially outwards at the source longitude and thus never encounters Earth. This transient *V* structure distorts the Earth-connected HMF away from an ideal Parker spiral^23^ configuration. Indeed, in the movie of this time period, inverted HMF can be clearly seen to form as a result of stream sheer^37^. In this TD model there are no discontinuous jumps between forecast conditions on consecutive days.

When the HUXt model is instead used as a series of independent SS solutions, the effect of this burst of fast wind is very different. The SS solution produced on 2024-12-13 only “knows” that there is a fast wind source just ahead of Earth, not how recently it appeared. Thus the SS model produces a co-rotating fast stream that extends out past 1 AU and (incorrectly) forecasts fast wind at Earth. Conversely, on 2024-12-14, when the fast stream is no longer present in the inner boundary conditions, the fast stream instantaneously disappears from the heliosphere. Throughout all SS runs, the Earth-connected HMF remains close to an ideal Parker spiral.

**Fig. 6:**
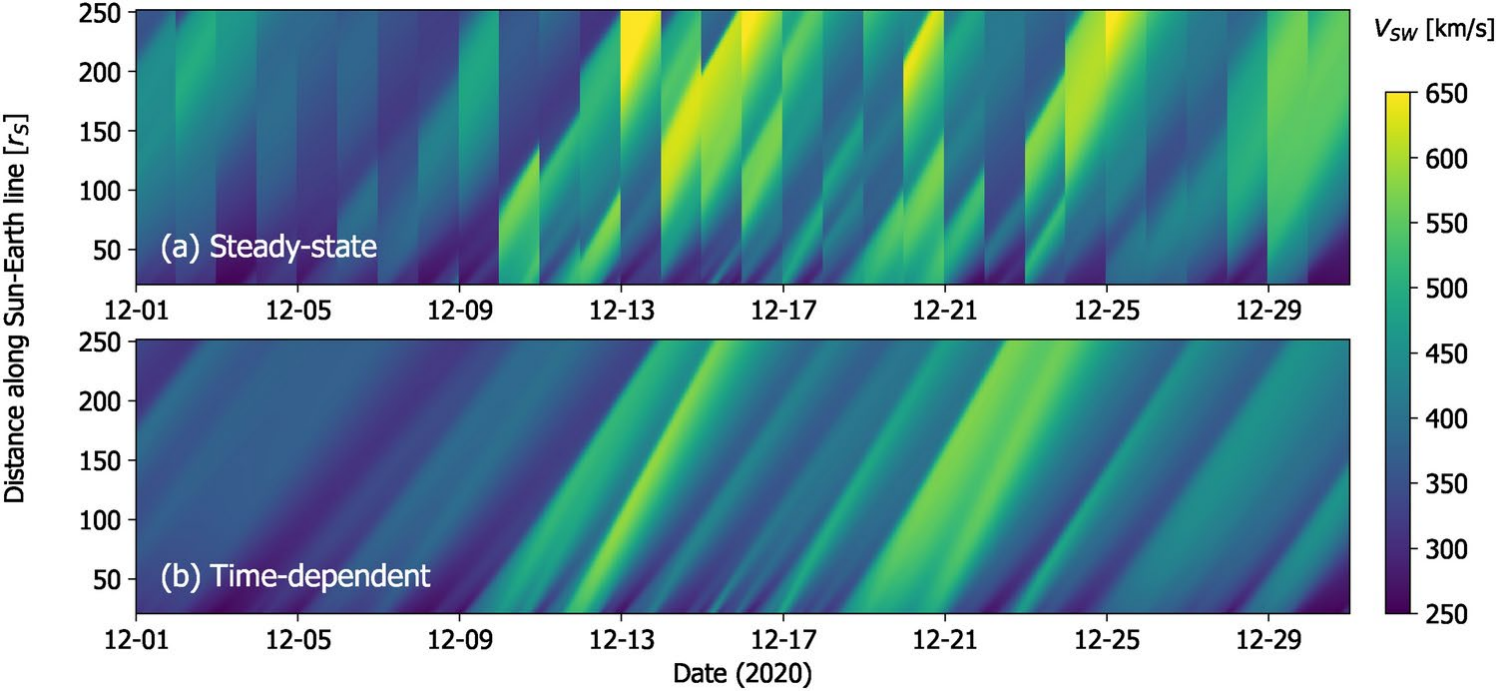
Plots of *V* as a function of distance along the Sun-Earth line and time for December 2020, from the HUXt solar wind model using daily-updated WSA solutions to the first ADAPT realisation of GONG magnetograms. Panel a shows the standard SS solution, whereas panel b shows the TD solution.

Figure 6 summarises *V* along the Sun-Earth line from SS and TD HUXt solutions over December 2020. The TD solution produces a physically consistent evolution of structures, with fast streams propagating outwards and producing sloping profiles somewhat reminiscent of “J-maps” constructed from Heliospheric Imager observations of propagating density structures in the solar wind^38^. Conversely, the SS solution shows a discontinuous change on a daily timescale as the model switches to a new WSA-GONG-ADAPT solution and throws away the information from the previous day.

The next section provides a more quantitative comparison of the SS and TD approaches when used in a forecast-like scenario over the whole of 2020.

### Statistical analysis

**Fig. 7:**
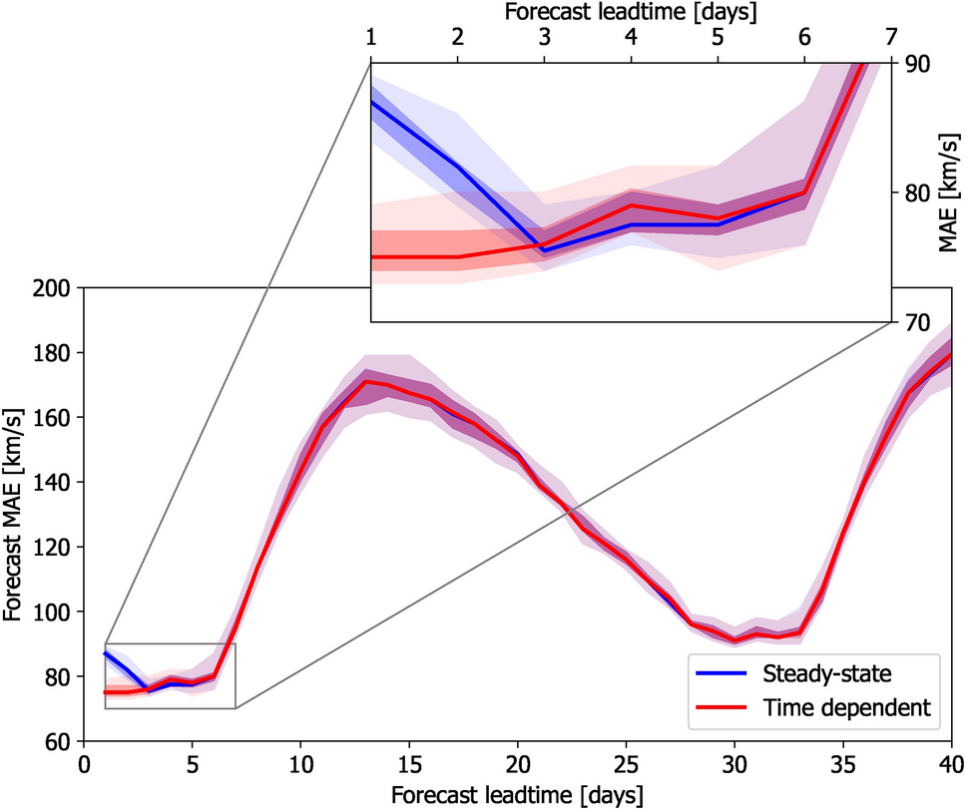
The mean-absolute-error (MAE) between forecast and observed *V* for a range of forecast lead times, evaluated over 2020. Solid lines lines show the medians of the HUXt ensembles from the 12 different WSA-GONG-ADAPT realisations. Shaded areas show the inter-quartile and full ranges of the ensemble. Blue lines/shading indicate SS solar-wind solutions, red lines/shading indicate TD solar-wind solutions.

The accuracy of solar-wind forecasts in near-Earth space is primarily determined by the accuracy of the coronal model that provides the inner-boundary condition. Nevertheless, it is still instructive to compare the SS and TD solar-wind model results with the observed *V* in order to understand how the treatment of the same inner-boundary conditions affects the forecast. Figure 7 shows how the mean-absolute-error (MAE) between the observed hourly *V* and the model estimates in near-Earth space varies with increasing forecast lead time. As reported by^24^, the SS forecast is less accurate at 1-day lead times than at 2- to 6-day lead times. The TD forecast shows a fairly constant MAE from 1- to 6-day lead times. The accuracy of the SS and TD forecasts is indistinguishable beyond 3-days lead time. This is expected, as once all the time-varying inner-boundary conditions have propagated past Earth, both the SS and TD approaches project forward in time by assuming the current inner-boundary conditions persist into the future.

On much longer timescales, both TD and SS approaches show a sharp increase in MAE beyond 7 days. This is a result of the source longitude for Earth-directed solar wind being behind the east limb of the Sun—and hence unobserved—at the time the forecast is issued. The MAE peaks around 13-days lead time, which is half a solar synodic rotation period, as that means the whole of the source hemisphere is unobserved at the forecast time. At longer lead times still, the MAE declines until around 27 days, when the same solar-wind source longitude was last facing Earth. This is the basis for 27-day recurrence forecasting^39–41^. Thus the gross behaviour of MAE in this plot can be primarily understood in terms of when the solar longitude of interest—which moves by approximately 13-degrees per day of forecast lead time—was last observed.

**Fig. 8:**
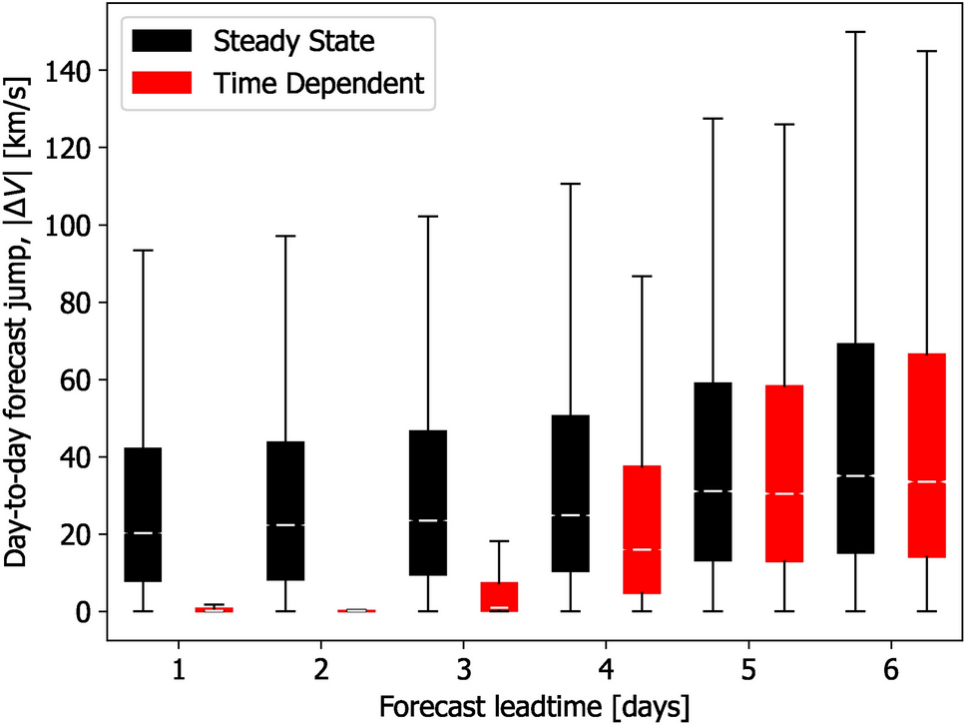
Box-and-whisker plots of forecast ‘jumpiness’ for a range of forecast lead times. Jumpiness is defined as the change in forecast *V* as the lead time reduces by 1 day. White lines show median values, boxes show interquartile ranges and thin lines show the full range. Black and red colours denote the SS and TD approaches, respectively.

Figure 8 summarises $|\Delta V|$, the absolute change in forecast *V* for a given day as new WSA-GONG-ADAPT solutions are introduced. For the TD forecasts, changes to the inner-boundary conditions take time to physically propagate out to Earth, meaning the forecasts for 1- to 3-day lead times remain relatively consistent as new observations are introduced. I.e. $\Delta V \approx 0$ for short lead times. Jumpiness in the TD forecasts occurs beyond 3–4 days lead time, as the forecasts effectively revert to SS, as all time-dependent information from the inner-boundary conditions has already propagated out past Earth.

Conversely, for SS forecasts, changes in the boundary conditions are instantaneously—and unphysically—transmitted to near-Earth space (and beyond). This means that the forecast *V* in near-Earth space ‘jumps’ significantly from day to day, even for short forecast lead times. To put these changes in ambient solar wind into context, using the HUXt model with a CME of speed 800 km s$^{-1}$, a change in ambient solar wind speed of 20, 40 and 100 km s$^{-1}$—the median, upper-quartile and full-range values of $\Delta V$ for 2-days lead time—changes the CME transit time by 3, 5.5, and 17 h, respectively.

**Fig. 9:**
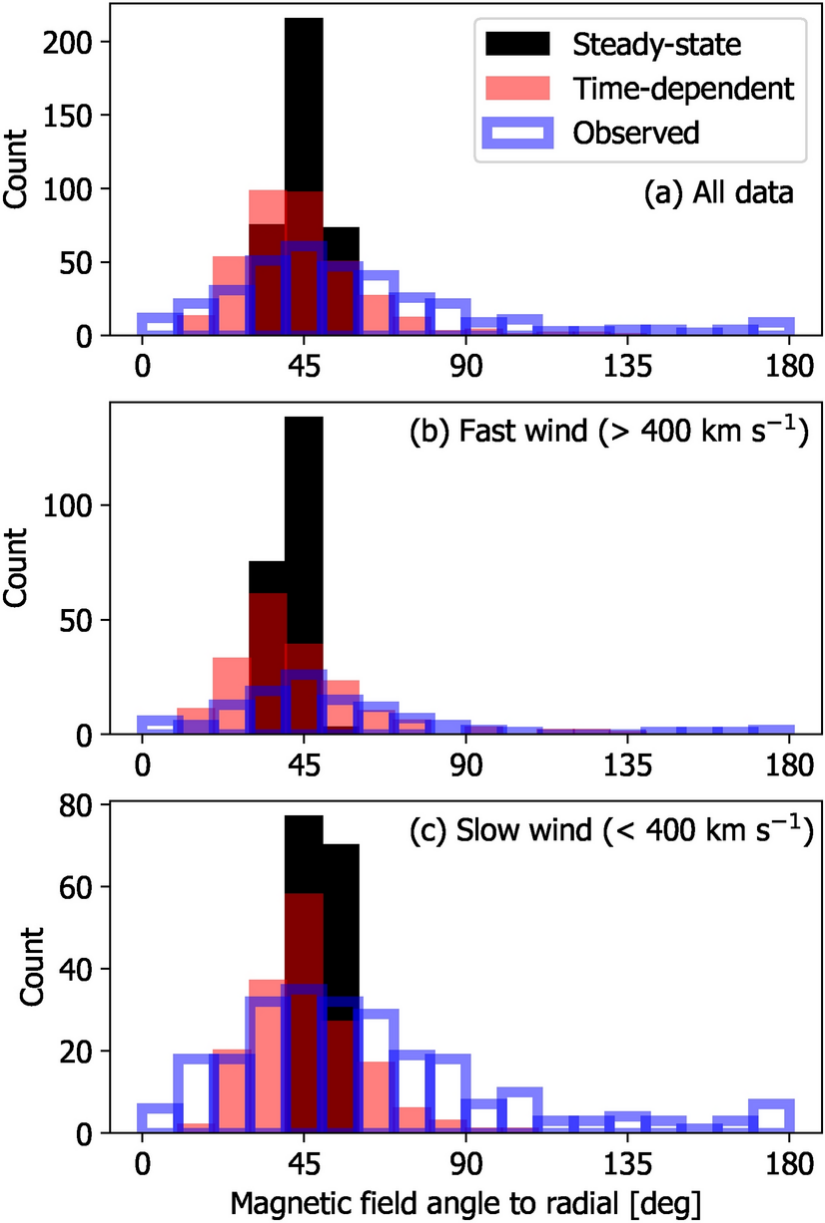
Histograms of the HMF angle to the radial direction in near-Earth space at 1-h resolution. Panel a shows the whole of 2020, panels b and c show the 2020 interval divided into fast and slow wind, respectively, using a threshold of 400 km s$^{-1}$. The observed distributions are shown in blue, with the SS and TD model solutions in black and red, respectively.

Finally, we consider the distortion of the HMF, particularly the Earth-connected HMF, from the ideal Parker spiral, as it is important for SEP forecasting. Unfortunately, the available observations mean that it is only possible to validate against observations with the HMF orientation at Earth. On average the HMF has long been observed to make an angle to the Earth-Sun line of around $45^{\circ }$^42^, with this angle reducing in fast wind and increasing in slow wind (e.g. Fig. 2 of^43^). But a great deal of scatter about that average direction is observed at the hourly time scale. Using the OMNI near-Earth observations for 2020, the blue outlines in Fig. 9 show these observed trends. Assuming the solar-wind flow streakline is equivalent to the HMF orientation (i.e. assuming the HMF moves passively with the solar-wind flow), we can compute the HMF angle from the HUXt model^33^. For the SS solution, there is very little scatter about the ideal Parker spiral angle. But for the TD solution, a much larger scatter is produced about this $45^\circ$ angle, as observed. The implications of these results are discussed below.

## Discussion

This study has compared steady-state (SS) and time-dependent (TD) approaches to modelling the solar wind, in both cases using the same daily-updated coronal model solutions as the model inner-boundary conditions.

Previous solar-wind model validation studies have noted the peculiar behaviour that forecasts based on the most recent observations are less accurate than forecasts produced three-to-four days previously. We have shown here that this is a direct result of SS solar-wind modelling and that this behaviour is removed by taking a TD approach. A case study showed how this is the result of SS modelling producing fast streams which instantly “appear” at large heliospheric distances, rather physically propagating from the Sun outwards. Of course, when forecasting only the ambient solar-wind conditions with an SS approach, this is not a major issue, as the 3-day ahead forecast can always be used in place of the most recent forecast. However, when forecasting CME arrival time at Earth, this is not practical, as a fast CME will encounter solar wind of a range of ages. Assuming coronal models can adequately capture the time evolution of the corona, a TD approach to ambient solar-wind modelling will provide a significant improvement to CME forecasting. This will be statistically tested in a future study.

Perhaps more importantly, the forecast accuracy/lead-time issue highlights that the SS approach is misrepresenting the solar-wind structure determined by the coronal models. Thus the current standard method for validation of coronal models may be flawed. The highlighted case study shows that even if a series of a coronal model solutions perfectly capture the evolution of the solar wind at 0.1 AU, SS solar-wind solutions would misrepresent the expected solar wind at 1 AU, where it is routinely compared with observations. This suggests an immediate need to move to time-dependent solar-wind modelling for coronal-model validation studies.

The lack of continuity between consecutive SS solar-wind solutions also means that the forecast for a given day can change dramatically as the forecast lead-time decreases. For short lead times of a few days, the SS forecast can change by up to 100 km/s. For typical CME speed of 800 km/s, this can change the predicted arrival time at Earth by around 17 h; around a third of the Sun-Earth travel time. Such forecast “jumpiness” is a known barrier to users being able to act decisively on the basis of a forecast^44^. Conversely, any changes at the inner boundary of the TD solar-wind model must physically propagate out through the heliosphere to 1 AU. This means that for lead times less than 3–4 days, there is very little change in the forecast with lead-time, providing a more actionable forecast.

The treatment of the inner-boundary conditions also affects the reconstructed heliospheric magnetic field (HMF), which is critical to solar energetic particle (SEP) propagation and forecasting at Earth^45^. On average, the HMF is observed to be well described by the Parker spiral configuration, with an average angle to the radial direction at Earth orbit of around 45$^\circ$. However, at the hourly to daily timescale, there is a great deal of variability around this average orientation. Furthermore, the HMF is often seen to become inverted from the expected orientation^46^, which has been attributed to solar-wind flow shear^37^ resulting from time-dependent solar wind flows. SS solar-wind modelling does produce stream-interaction regions which slightly distort the HMF from the ideal Parker spiral direction, but the variability in the HMF angle at Earth is markedly lower than observed, and HMF inversions are not produced. Conversely, the TD approach naturally produces HMF inversions (see also^10,19^) and a much closer match to the observed distribution of HMF angles at Earth. The non-Parker HMF produces longer propagation paths for SEPs, which is expected from theory and observations^47^. The implications of TD modelling for improved SEP forecasting will form the basis of a future study.

Finally, we note that while the results presented here have been generated using the HUXt solar wind model, they should be generally applicable to all solar-wind models. Similarly, the TD approach is relatively trivial to implement, regardless of solar-wind model. It is hoped the advantages of the TD approach demonstrated here can provide the impetus to move beyond the SS approach that has been used for the last two decades.

## Supplementary Information

Supplementary Information.

## Data Availability

All code and data are available at https://github.com/University-of-Reading-Space-Science/time-dependent_solar_wind.
